# Supplementary material for: A Type II-B Cas9 nuclease with minimized off-targets and reduced chromosomal translocations in vivo
Source: Nat Commun. 2023 Sep 6;14:5474. doi: 10.1038/s41467-023-41240-7 (PMC10482872; doi:10.1038/s41467-023-41240-7)
Supplement: Supplementary file 1 — Supplementary Information [file 41467_2023_41240_MOESM1_ESM.pdf]

A Type II-B Cas9 nuclease with minimized off-targets and reduced chromosomal translocations *in vivo*.

Bestas et al. Supplementary Figure 1

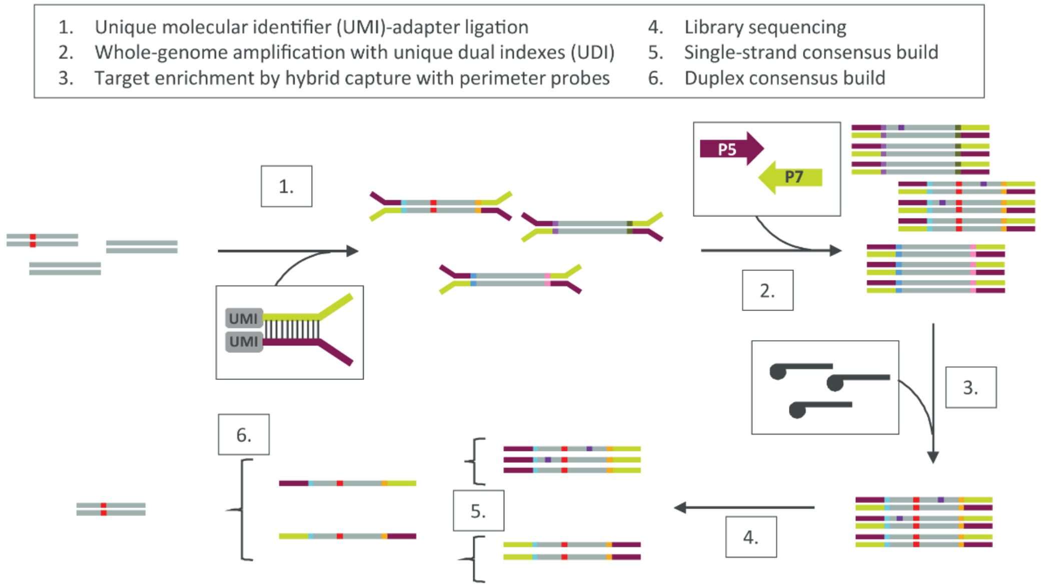

Supplementary Figure 1. Overview Duplex-Seq workflow.

Schematic depiction of Duplex-Seq workflow. After shearing of genomic DNA, UMI-labelled adapters are ligated to the DNA fragments (1), followed by whole-genome amplification with UDIs (2). Enrichment of target sites from this unbiased and unenriched library is performed by hybrid capture with four perimeter probes per site (3). After library sequencing (4), a single-strand consensus sequence is built from replicate reads of each strand (5), followed by duplex-consensus sequence generation (6). The double consensus build facilitates superior sequencing error elimination (purple squares) compared to standard NGS analysis, enabling a more sensitive identification of actual mutations (red squares). Furthermore, probe-based target enrichment enables analysis of translocations. UMIs are depicted as differently coloured boxes at the end of each adapter. UMI=unique molecular identifier; UDIs=unique dual indexes.

Bestas et al. Supplementary Figure 2

	VIVO (2018): Amplicon-sequencing						
	SpCas9			Control			
CAGGTTCCATGGGATGCTCTNGG	1	2	3	1	2	3	
••••••••••••••••••••G••	46.1	41.6	37.0	<0.1	<0.1	<0.1	*mPcsk9*
•••••••••C••••••••••G••	39.6	37.7	31.0	<0.1	<0.1	<0.1	*hPCSK9*
•••T•••••••••G••••••T••	<0.1	<0.1	<0.1	<0.1	<0.1	<0.1	chr3:37482936-37482958
•••T••••••••A•••T•••A••	<0.1	<0.1	<0.1	<0.1	<0.1	<0.1	chr5:98601866-98601888
•••C••••••A•••••••G•A••	<0.1	<0.1	<0.1	<0.1	<0.1	<0.1	chr6:6533816-6533838
•••A•••••••T••••••T•A••	<0.1	<0.1	<0.1	<0.1	<0.1	<0.1	chr16:8681073-8681095
•••CA•••••••••••G•••G••	<0.1	<0.1	<0.1	<0.1	<0.1	<0.1	chr15:94999993-95000015
••••••T•••••AG••••••A••	<0.1	<0.1	<0.1	<0.1	<0.1	<0.1	chr4:93574282-93574304
G•••G•••••••••••••••AA•	<0.1	<0.1	<0.1	<0.1	<0.1	<0.1	chr8:26866402-26866424
••••••AG••A•••••••••C••	<0.1	<0.1	<0.1	<0.1	<0.1	<0.1	chr15:74426204-74426226
••••G••••••••••••C••GA•	<0.1	<0.1	<0.1	<0.1	<0.1	<0.1	chr18:64586066-64586088 **(OT-1)**
•••••G••••••C•••••••CA•	<0.1	<0.1	<0.1	<0.1	<0.1	<0.1	chr8:60461130-60461152
••••••TT••••T•••••••A••	<0.1	<0.1	<0.1	<0.1	<0.1	<0.1	chr16:12263585-12263607
•T••••••••••••G••••AA••	<0.1	<0.1	<0.1	<0.1	<0.1	<0.1	chr7:140152795-140152817
•••••••A••••••G•••••G•A	<0.1	<0.1	<0.1	<0.1	<0.1	<0.1	chr12:104434800-104434822
••••••••T••T••G•••••T••	<0.1	<0.1	<0.1	<0.1	<0.1	<0.1	chr15:78106077-78106099
••••••G•C•••••••••••TT•	<0.1	<0.1	<0.1	<0.1	<0.1	<0.1	chr16:18132431-18132453
•••A•••••••••••••A••GT•	<0.1	<0.1	<0.1	<0.1	<0.1	<0.1	chr16:90528829-90528851
•••••••••C•••C•••••CT••	<0.1	<0.1	<0.1	<0.1	<0.1	<0.1	chr16:96569049-96569071
•T•••G••C•••••••••••G••	<0.1	<0.1	<0.1	<0.1	<0.1	<0.1	chr17:33501738-33501760
••••••T•••••T•••G•••A••	<0.1	<0.1	<0.1	<0.1	<0.1	<0.1	chr3:14980373-14980395
•••••••••G•••T••••••CA•	<0.1	<0.1	<0.1	<0.1	<0.1	<0.1	chr4:89194529-89194551
••••••••••••••••AA•AT••	<0.1	<0.1	<0.1	<0.1	<0.1	<0.1	chr7:54903019-54903041
•••••G••T••••••T••••T••	<0.1	<0.1	<0.1	<0.1	<0.1	<0.1	chr9:123273285-123273307
AT••••••••A••••••••AT••	<0.1	<0.1	<0.1	<0.1	<0.1	<0.1	chr14:49433410-49433432 **(OT-2)**
•••A•C••T•••••••••••CA•	<0.1	<0.1	<0.1	<0.1	<0.1	<0.1	chr16:33683431-33683453
••••A••••GA•••••••••GA•	<0.1	<0.1	<0.1	<0.1	<0.1	<0.1	chr3:66104632-66104654
TC••••••••T•••••••••CA•	<0.1	<0.1	<0.1	<0.1	<0.1	<0.1	chr14:90472334-90472356
••A••CT••••••••••A••T••	<0.1	<0.1	<0.1	<0.1	<0.1	<0.1	chr2:7048085-7048107
•••AC••••A•••••••••AA••	<0.1	<0.1	<0.1	<0.1	<0.1	<0.1	chr6:117133010-117133032
••••AA•••A•••••••C••G••	<0.1	<0.1	<0.1	<0.1	<0.1	<0.1	chr13:110714780-110714802
••••A•••••AC••••••••AA•	<0.1	<0.1	<0.1	<0.1	<0.1	<0.1	chr2:155742625-155742647
T•T•G•••••••••••••G•A••	<0.1	<0.1	<0.1	<0.1	<0.1	<0.1	chr11:3308538-3308560
••••CC•••••••GA•••••T••	<0.1	<0.1	<0.1	<0.1	<0.1	<0.1	chrX:12131461-12131483 **(OT-3)**
GCT•••••T•••••••••••G••	<0.1	<0.1	<0.1	<0.1	<0.1	<0.1	chr2:166541037-166541059 **(OT-4)**
A••••G••••••••••T•••GA•	<0.1	<0.1	<0.1	<0.1	<0.1	<0.1	chr11:40201599-40201621
•T••CC••••••••••••••TA•	<0.1	<0.1	<0.1	<0.1	<0.1	<0.1	chr2:35899961-35899983
•T••••••T••••••A•••AA••	<0.1	<0.1	<0.1	<0.1	<0.1	<0.1	chr5:102779883-102779905
•TCT••••••••••••••••GA•	<0.1	<0.1	<0.1	<0.1	<0.1	<0.1	chr11:106016683-106016705
••••A•A••••A•••••C••T••	<0.1	<0.1	<0.1	<0.1	<0.1	<0.1	chr6:30643921-30643943
TGTT••••••••••••••••T••	<0.1	<0.1	<0.1	<0.1	<0.1	<0.1	chr9:102025045-102025067
A••C••••••A•••••••••C•T	<0.1	<0.1	<0.1	<0.1	<0.1	<0.1	chr4:90867320-90867342
•CA••A•••••A••••••••T••	3.5	3.3	3.5	3.8	3.7	3.9	chr15:4878203-4878225
•CA•A•••••••••••••••GA•	<0.1	<0.1	<0.1	<0.1	<0.1	<0.1	chrX:97929779-97929801
•••••G•T••AA••••••••G••	<0.1	<0.1	<0.1	<0.1	<0.1	<0.1	chr16:95293068-95293090
G••••C••T•••••••••G•A••	<0.1	<0.1	<0.1	<0.1	<0.1	<0.1	chr6:119072074-119072096
••••GG••••••••AA••••G••	<0.1	<0.1	<0.1	<0.1	<0.1	<0.1	chr8:72352454-72352476
••••••••CGA•••••••••CA•	<0.1	<0.1	<0.1	<0.1	<0.1	<0.1	chr18:81581575-81581597
T•••••••••A••••••CA•T••	<0.1	<0.1	<0.1	<0.1	<0.1	<0.1	chr1:96553037-96553059
G••••••••A••C••••C••G••	<0.1	<0.1	<0.1	<0.1	<0.1	<0.1	chr10:112266314-112266336
•••C•C•••••••G•••••AG••	<0.1	<0.1	<0.1	<0.1	<0.1	<0.1	chr10:68654995-68655017
••T••••••••••TA••C••A••	<0.1	<0.1	<0.1	<0.1	<0.1	<0.1	chr16:86149241-86149263
GT•A••••••A•••••••••GT•	<0.1	<0.1	<0.1	<0.1	<0.1	<0.1	chr9:94884378-94884400
TGT•••••••••A•••••T•T••	<0.1	<0.1	<0.1	<0.1	<0.1	<0.1	chr2:64822488-64822510
•TA•••••••A••G•••C••T••	<0.1	<0.1	<0.1	<0.1	<0.1	<0.1	chr6:126638347-126638369
TTT•••••••••AT••••••T••	<0.1	<0.1	<0.1	<0.1	<0.1	<0.1	chr3:73871325-73871347
AGT••••••••A••G•••••T••	<0.1	<0.1	<0.1	<0.1	<0.1	<0.1	chr7:73364968-73364990
•TTCC•••••••••••••G•A••	<0.1	<0.1	<0.1	<0.1	<0.1	<0.1	chr6:71131071-71131093
AGCC••••••••••A•••••A••	<0.1	<0.1	<0.1	<0.1	<0.1	<0.1	chr3:125637095-125637117
TG••••T•••A•••A•••••A••	<0.1	<0.1	<0.1	<0.1	<0.1	<0.1	chr3:96132216-96132238 **(OT-5)**
••••G•A•C•••A•••T•••G••	<0.1	<0.1	<0.1	<0.1	<0.1	<0.1	chr15:36419958-36419980
•••AG••••••AA•A•••••G••	<0.1	<0.1	<0.1	<0.1	<0.1	<0.1	chr6:101076190-101076212
ACA•G••T••C•••••••••G••	<0.1	<0.1	<0.1	<0.1	<0.1	<0.1	chr6:52990895-52990917
TTTA•C••••••••••••••GA•	<0.1	<0.1	<0.1	<0.1	<0.1	<0.1	chr4:71181962-71181984
••A•GCT•C•••••••••T•A••	<0.1	<0.1	<0.1	<0.1	<0.1	<0.1	chr2:15574929-15574951
ACT••A•••••A••A•••••T••	<0.1	<0.1	<0.1	<0.1	<0.1	<0.1	chr4:34075690-34075712
TT•••C•••C••••••TA••T••	<0.1	<0.1	<0.1	<0.1	<0.1	<0.1	chr3:126805179-126805201
AGA•GC••••••C•••••••G••	<0.1	<0.1	<0.1	<0.1	<0.1	<0.1	chr11:108975678-108975700
ACT••••T••A•••A•••••A••	<0.1	<0.1	<0.1	<0.1	<0.1	<0.1	chr1:32520795-32520817
TCT•••T•••A•••••••••GT•	<0.1	<0.1	<0.1	<0.1	<0.1	<0.1	chr16:25412869-25412891
ATA•GC••••A••••••C••A••	<0.1	<0.1	<0.1	<0.1	<0.1	<0.1	chr2:3973697-3973719
TCA•G•••CA•••••••C••T••	<0.1	<0.1	<0.1	<0.1	<0.1	<0.1	chr12:108405696-108405718
GCCA•C••••A•••••••••AA•	<0.1	<0.1	<0.1	<0.1	<0.1	<0.1	chr4:129286756-129286778
GCAAG•T•••••••••••••AA•	<0.1	<0.1	<0.1	<0.1	<0.1	<0.1	chr8:14578550-14578572
ACTC••••••AA••••••••GA•	<0.1	<0.1	<0.1	<0.1	<0.1	<0.1	chr16:85838458-85838480
•••A••••-•A•••••••••GA•	<0.1	<0.1	<0.1	<0.1	<0.1	<0.1	chr12:111733441-111733462
•••••••••-••••••••••G•A	<0.1	<0.1	<0.1	<0.1	<0.1	<0.1	chr9:118783391-118783412
••••C•••-•A•••••••T•G••	<0.1	<0.1	<0.1	<0.1	<0.1	<0.1	chr17:8243138-8243159
•••C••••-••A•••••C••A••	<0.1	<0.1	<0.1	<0.1	<0.1	<0.1	chr4:108892830-108892851
T•••••••-•••••GA••••A••	<0.1	<0.1	<0.1	<0.1	<0.1	<0.1	chr2:58419837-58419858

Supplementary Figure 2. Extended table *in vivo* amplicon sequencing data.

Extended table from Figure 1C showing gene editing efficiency for all target sites assayed by multiplexed amplicon sequencing followed by CRISPResso2 analysis. Heatmaps present editing percentages for the on-target sites (highlighted in green) and 78 off-target sites (highlighted in magenta). Amplicon sequencing was performed with n=3 biologically independent mice per group (1, 2, 3) using genomic DNA isolated from the liver of mice treated with adenoviral vector expressing either SpCas9 plus gMH sgRNA or SpCas9-GFP as a control. Values show non-normalized editing data as percent indel-containing reads. All values < 0.1% fall below the detection limit of amplicon sequencing and are considered as non-specific/background.

Bestas et al. Supplementary Figure 3

	Duplex-sequencing (2022)									
	SpCas9			PsCas9			Control			
CAGGTTCCATGGGATGCTCTNGG	1	2	3	1	2	3	1	2	3	
••••••••••••••••••••G••	41.62	38.35	33.82	38.49	26.05	28.16	<0.01	<0.01	<0.01	*mPcsk9*
•••••••••C••••••••••G••	39.14	39.46	31.59	26.12	19.11	18.57	<0.01	<0.01	<0.01	*hPCSK9*
•••T•••••••••G••••••T••	<0.01	<0.01	<0.01	<0.01	<0.01	<0.01	<0.01	<0.01	<0.01	chr3:37482936-37482958
•••T••••••••A•••T•••A••	<0.01	<0.01	<0.01	<0.01	<0.01	<0.01	<0.01	<0.01	<0.01	chr5:98601866-98601888
•••C••••••A•••••••G•A••	<0.01	<0.01	<0.01	<0.01	<0.01	<0.01	<0.01	<0.01	<0.01	chr6:6533816-6533838
•••A•••••••T••••••T•A••	<0.01	<0.01	<0.01	<0.01	<0.01	<0.01	<0.01	<0.01	<0.01	chr16:8681073-8681095
•••CA•••••••••••G•••G••	<0.01	<0.01	<0.01	<0.01	<0.01	<0.01	<0.01	<0.01	<0.01	chr15:94999993-95000015
••••••T•••••AG••••••A••	<0.01	<0.01	<0.01	<0.01	<0.01	<0.01	<0.01	<0.01	<0.01	chr4:93574282-93574304
G•••G•••••••••••••••AA•	<0.01	<0.01	<0.01	<0.01	<0.01	<0.01	<0.01	<0.01	<0.01	chr8:26866402-26866424
••••••AG••A•••••••••C••	<0.01	<0.01	<0.01	<0.01	<0.01	<0.01	<0.01	<0.01	<0.01	chr15:74426204-74426226
••••••••••A••TG•••••G••	<0.01	<0.01	<0.01	<0.01	<0.01	<0.01	<0.01	<0.01	<0.01	chr14:60784833-60784855
••••G••••••••••••C••GA•	0.02	<0.01	<0.01	<0.01	<0.01	<0.01	<0.01	<0.01	<0.01	chr18:64586066-64586088
•••••G••••••C•••••••CA•	<0.01	<0.01	<0.01	<0.01	<0.01	<0.01	<0.01	<0.01	<0.01	chr8:60461130-60461152
••••••TT••••T•••••••A••	<0.01	<0.01	<0.01	<0.01	<0.01	<0.01	<0.01	<0.01	<0.01	chr16:12263585-12263607
•T••••••••••••G••••AA••	<0.01	<0.01	<0.01	<0.01	<0.01	<0.01	<0.01	<0.01	<0.01	chr7:140152795-140152817
•••••••A••••••G•••••G•A	<0.01	<0.01	<0.01	<0.01	<0.01	<0.01	<0.01	<0.01	<0.01	chr12:104434800-10443482
••••••••T••T••G•••••T••	<0.01	<0.01	<0.01	<0.01	<0.01	<0.01	<0.01	<0.01	<0.01	chr15:78106077-78106099
••••••G•C•••••••••••TT•	<0.01	<0.01	<0.01	<0.01	<0.01	<0.01	<0.01	<0.01	<0.01	chr16:18132431-18132453
•••A•••••••••••••A••GT•	<0.01	<0.01	<0.01	<0.01	<0.01	<0.01	<0.01	<0.01	<0.01	chr16:90528829-90528851
•••••••••C•••C•••••CT••	<0.01	<0.01	<0.01	<0.01	<0.01	<0.01	<0.01	<0.01	<0.01	chr16:96569049-96569071
•T•••G••C•••••••••••G••	<0.01	<0.01	<0.01	<0.01	<0.01	<0.01	<0.01	<0.01	<0.01	chr17:33501738-33501760
••••••T•••••T•••G•••A••	<0.01	<0.01	<0.01	<0.01	<0.01	<0.01	<0.01	<0.01	<0.01	chr3:14980373-14980395
•••••••••G•••T••••••CA•	<0.01	<0.01	<0.01	<0.01	<0.01	<0.01	<0.01	<0.01	<0.01	chr4:89194529-89194551
••••••••••••••••AA•AT••	<0.01	<0.01	<0.01	<0.01	<0.01	<0.01	<0.01	<0.01	<0.01	chr7:54903019-54903041
•••••G••T••••••T••••T••	<0.01	<0.01	<0.01	<0.01	<0.01	<0.01	<0.01	<0.01	<0.01	chr9:123273285-123273307
AT••••••••A••••••••AT••	<0.01	<0.01	0.01	<0.01	<0.01	<0.01	<0.01	<0.01	<0.01	chr14:49433410-49433432
•••A•C••T•••••••••••CA•	<0.01	<0.01	<0.01	<0.01	<0.01	<0.01	<0.01	<0.01	<0.01	chr16:33683431-33683453
••••A••••GA•••••••••GA•	<0.01	<0.01	<0.01	<0.01	<0.01	<0.01	<0.01	<0.01	<0.01	chr3:66104632-66104654
••A••CT••••••••••A••T••	<0.01	<0.01	<0.01	<0.01	<0.01	<0.01	<0.01	<0.01	<0.01	chr2:7048085-7048107
•••AC••••A•••••••••AA••	<0.01	<0.01	<0.01	<0.01	<0.01	<0.01	<0.01	<0.01	<0.01	chr6:117133010-11713303
••••A•••••AC••••••••AA•	<0.01	<0.01	<0.01	<0.01	<0.01	<0.01	<0.01	<0.01	<0.01	chr2:155742625-15574264
T•T•G•••••••••••••G•A••	<0.01	<0.01	<0.01	<0.01	<0.01	<0.01	<0.01	<0.01	<0.01	chr11:3308538-3308560
••••CC•••••••GA•••••T••	0.01	<0.01	<0.01	<0.01	<0.01	<0.01	<0.01	<0.01	<0.01	chrX:12131461-12131483
GCT•••••T•••••••••••G••	0.01	0.04	0.02	<0.01	<0.01	<0.01	<0.01	<0.01	<0.01	chr2:166541037-16654105
A••••G••••••••••T•••GA•	<0.01	<0.01	<0.01	<0.01	<0.01	<0.01	<0.01	<0.01	<0.01	chr11:40201599-40201621
•T••CC••••••••••••••TA•	<0.01	<0.01	<0.01	<0.01	<0.01	<0.01	<0.01	<0.01	<0.01	chr2:35899961-35899983
•T••••••T••••••A•••AA••	<0.01	<0.01	<0.01	<0.01	<0.01	<0.01	<0.01	<0.01	<0.01	chr5:102779883-10277990
•TCT••••••••••••••••GA•	<0.01	<0.01	<0.01	<0.01	<0.01	<0.01	<0.01	<0.01	<0.01	chr11:106016683-1060167
••••A•A••••A•••••C••T••	<0.01	<0.01	<0.01	<0.01	<0.01	<0.01	<0.01	<0.01	<0.01	chr6:30643921-30643943
TGTT••••••••••••••••T••	<0.01	<0.01	<0.01	<0.01	<0.01	<0.01	<0.01	<0.01	<0.01	chr9:102025045-102025067
A••C••••••A•••••••••C•T	<0.01	<0.01	<0.01	<0.01	<0.01	<0.01	<0.01	<0.01	<0.01	chr4:90867320-90867342
•CA•A•••••••••••••••GA•	<0.01	<0.01	<0.01	<0.01	<0.01	<0.01	<0.01	<0.01	<0.01	chrX:97929779-97929801
•••••G•T••AA••••••••G••	<0.01	<0.01	<0.01	<0.01	<0.01	<0.01	<0.01	<0.01	<0.01	chr16:95293068-95293090
G••••C••T•••••••••G•A••	<0.01	<0.01	<0.01	<0.01	<0.01	<0.01	<0.01	<0.01	<0.01	chr6:119072074-11907209
••••GG••••••••AA••••G••	<0.01	<0.01	<0.01	<0.01	<0.01	<0.01	<0.01	<0.01	<0.01	chr8:72352454-72352476
••••••••CGA•••••••••CA•	<0.01	<0.01	<0.01	<0.01	<0.01	<0.01	<0.01	<0.01	<0.01	chr18:81581575-81581597
T•••••••••A••••••CA•T••	<0.01	<0.01	<0.01	<0.01	<0.01	<0.01	<0.01	<0.01	<0.01	chr1:96553037-96553059
G••••••••A••C••••C••G••	<0.01	<0.01	<0.01	<0.01	<0.01	<0.01	<0.01	<0.01	<0.01	chr10:112266314-11226633
•••C•C•••••••G•••••AG••	<0.01	<0.01	<0.01	<0.01	<0.01	<0.01	<0.01	<0.01	<0.01	chr10:68654995-68655017
••T••••••••••TA••C••A••	<0.01	<0.01	<0.01	<0.01	<0.01	<0.01	<0.01	<0.01	<0.01	chr16:86149241-86149263
GT•A••••••A•••••••••GT•	<0.01	<0.01	<0.01	<0.01	<0.01	<0.01	<0.01	<0.01	<0.01	chr9:94884378-94884400
TGT•••••••••A•••••T•T••	<0.01	<0.01	<0.01	<0.01	<0.01	<0.01	<0.01	<0.01	<0.01	chr2:64822488-64822510
•TA•••••••A••G•••C••T••	<0.01	<0.01	<0.01	<0.01	<0.01	<0.01	<0.01	<0.01	<0.01	chr6:126638347-12663836
TTT•••••••••AT••••••T••	<0.01	<0.01	<0.01	<0.01	<0.01	<0.01	<0.01	<0.01	<0.01	chr3:73871325-73871347
AGT••••••••A••G•••••T••	<0.01	<0.01	<0.01	<0.01	<0.01	<0.01	<0.01	<0.01	<0.01	chr7:73364968-73364990
•TTCC•••••••••••••G•A••	<0.01	<0.01	<0.01	<0.01	<0.01	<0.01	<0.01	<0.01	<0.01	chr6:71131071-71131093
AGCC••••••••••A•••••A••	<0.01	<0.01	<0.01	<0.01	<0.01	<0.01	<0.01	<0.01	<0.01	chr3:125637095-12563711
TG••••T•••A•••A•••••A••	<0.01	<0.01	0.02	<0.01	<0.01	<0.01	<0.01	<0.01	<0.01	chr3:96132216-96132238
••••G•A•C•••A•••T•••G••	<0.01	<0.01	<0.01	<0.01	<0.01	<0.01	<0.01	<0.01	<0.01	chr15:36419958-36419980
•••AG••••••AA•A•••••G••	<0.01	<0.01	<0.01	<0.01	<0.01	<0.01	<0.01	<0.01	<0.01	chr6:101076190-10107621
ACA•G••T••C•••••••••G••	<0.01	<0.01	<0.01	<0.01	<0.01	<0.01	<0.01	<0.01	<0.01	chr6:52990895-52990917
TTTA•C••••••••••••••GA•	<0.01	<0.01	<0.01	<0.01	<0.01	<0.01	<0.01	<0.01	<0.01	chr4:71181962-71181984
••A•GCT•C•••••••••T•A••	<0.01	<0.01	<0.01	<0.01	<0.01	<0.01	<0.01	<0.01	<0.01	chr2:15574929-15574951
ACT••A•••••A••A•••••T••	<0.01	<0.01	<0.01	<0.01	<0.01	<0.01	<0.01	<0.01	<0.01	chr4:34075690-34075712
TT•••C•••C••••••TA••T••	<0.01	<0.01	<0.01	<0.01	<0.01	<0.01	<0.01	<0.01	<0.01	chr3:126805179-12680520
AGA•GC••••••C•••••••G••	<0.01	<0.01	<0.01	<0.01	<0.01	<0.01	<0.01	<0.01	<0.01	chr11:108975678-1089757
ACT••••T••A•••A•••••A••	<0.01	<0.01	<0.01	<0.01	<0.01	<0.01	<0.01	<0.01	<0.01	chr1:32520795-32520817
TCT•••T•••A•••••••••GT•	<0.01	<0.01	<0.01	<0.01	<0.01	<0.01	<0.01	<0.01	<0.01	chr16:25412869-25412891
ATA•GC••••A••••••C••A••	<0.01	<0.01	<0.01	<0.01	<0.01	<0.01	<0.01	<0.01	<0.01	chr2:3973697-3973719
TCA•G•••CA•••••••C••T••	<0.01	<0.01	<0.01	<0.01	<0.01	<0.01	<0.01	<0.01	<0.01	chr12:108405696-10840571
GCCA•C••••A•••••••••AA•	<0.01	<0.01	<0.01	<0.01	<0.01	<0.01	<0.01	<0.01	<0.01	chr4:129286756-12928677
GCAAG•T•••••••••••••AA•	<0.01	<0.01	<0.01	<0.01	<0.01	<0.01	<0.01	<0.01	<0.01	chr8:14578550-14578572
•••A••••-•A•••••••••GA•	<0.01	<0.01	<0.01	<0.01	<0.01	<0.01	<0.01	<0.01	<0.01	chr12:111733441-11173346
•••••••••-••••••••••G•A	<0.01	<0.01	<0.01	<0.01	<0.01	<0.01	<0.01	<0.01	<0.01	chr9:118783391-11878341
••••C•••-•A•••••••T•G••	<0.01	<0.01	<0.01	<0.01	<0.01	<0.01	<0.01	<0.01	<0.01	chr17:8243138-8243159
•••C••••-••A•••••C••A••	<0.01	<0.01	<0.01	<0.01	<0.01	<0.01	<0.01	<0.01	<0.01	chr4:108892830-108892851
T•••••••-•••••GA••••A••	<0.01	<0.01	<0.01	<0.01	<0.01	<0.01	<0.01	<0.01	<0.01	chr2:58419837-58419858

Supplementary Figure 3. Extended table *in vivo* Duplex-Seq data.

Extended table from Figures 1B and 4C showing gene editing efficiency for all on/off-target sites assayed by Duplex-Seq followed by CRISPResso2 analysis. Heatmaps present editing percentages for the on-target sites (highlighted in green) and 75 off-target sites (highlighted in magenta). Duplex-Seq was performed with n=3 biologically independent mice per group (1, 2, 3) using genomic DNA isolated from the liver of mice treated with adenoviral vector expressing sgRNA gMH together with SpCas9 or PsCas9. Adenoviral vector encoding SpCas9 without a sgRNA was used as a control. Values show non-normalized editing data as percent indel-containing reads. The detection limit reached in this experimental set-up was 0.01%. All Duplex-Seq experiments were performed in parallel in the same experiment.

Bestas et al. Supplementary Figure 4

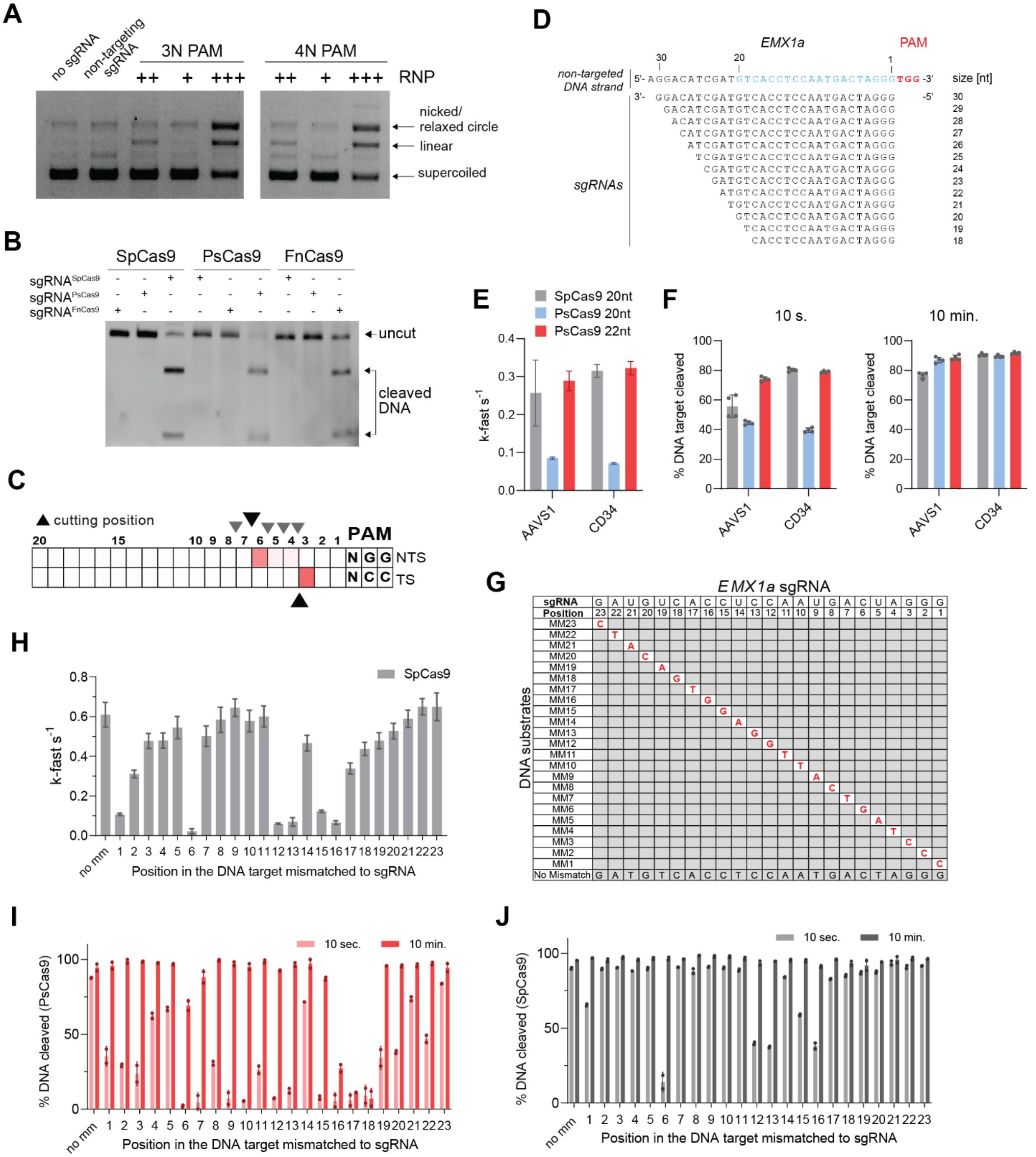

Supplementary Figure 4. Extended characterization of PsCas9, an RNA-guided DNA nuclease.

a. Gel electrophoresis analysis of 3 and 4 base pair randomized PAM sequence containing plasmid libraries (3N, 4N) cut with three different doses of RNP complex: 3 nM(+), 30 nM(++), 300 nM (+++). The first two lanes show plasmid libraries without RNP treatment as a control. Representative gel of at least two independent experiments.

b. Gel electrophoresis of *in vitro* cleaved or uncut DNA products after incubation with a nuclease-specific crRNA:tracrRNA duplex. Each combination for SpCas9, PsCas9 and FnCas9 were assessed individually. Representative gel of at least two independent experiments.

c. PsCas9 cleavage pattern extracted from the sequenced PAM plasmid library (4N). Heat map shows the calculated frequent (black arrows) and less-frequent (grey arrows) cleavage sites on target-strand (TS) and non-target strand (NTS).

d. Spacer targeting *EMX1a* with varying length from 18 to 30 nucleotides which were used in *in vitro* cleavage assays to determine the ideal configuration for PsCas9. The preferred spacer length of SpCas9 (20 bp) is highlighted in blue.

e, f. Plots showing the reaction rate (e) and the fraction (f) of *AAVSI* and *CD34* DNA substrate digested by SpCas9 and PsCas9 over time (10 sec and 10min) in the *in vitro* DNA cleavage assay. The amount of substrate digested was calculated using local integral fluorescent of capillary electrophoresis traces and fitted to the bi-exponential decay function. Reaction rate is presented as k_fast (kf). While SpCas9 was loaded with 20-nt spacer, PsCas9 was loaded with either 20-nt or 22-nt long spacers targeting depicted sites. Data are presented as mean ± SD, n=4 (all experimental timepoints in Source Data).

g. The *EMX1a* sgRNA sequence and DNA substrates that were used in Figure 2H and Supplementary Figure 4H-J to determine DNA cleavage rates.

h. In vitro DNA cleavage rates by wild-type SpCas9 with a mismatch at every single base position along the protospacer (see Supplementary Figure 4G). Mismatch positions are labelled in 3’ to 5’ direction, with the position 1 being directly upstream PAM. Reaction rate is presented as k_fast (kf). Data are presented as mean ± SD, n=2 (all experimental timepoints in Source Data).

i-j. In vitro the fraction of cleaved DNA of *EMX1a* by PsCas9 (i) and SpCas9 (j) with a mismatch at every single base position along the protospacer (see Figure 2H and Supplementary Figure 4G). Mismatch positions are labelled in 3’ to 5’ direction, with Position 1 being directly upstream PAM. Data are presented as mean ± SD, n=2 (all experimental timepoints in Source Data).

Bestas et al. Supplementary Figure 5

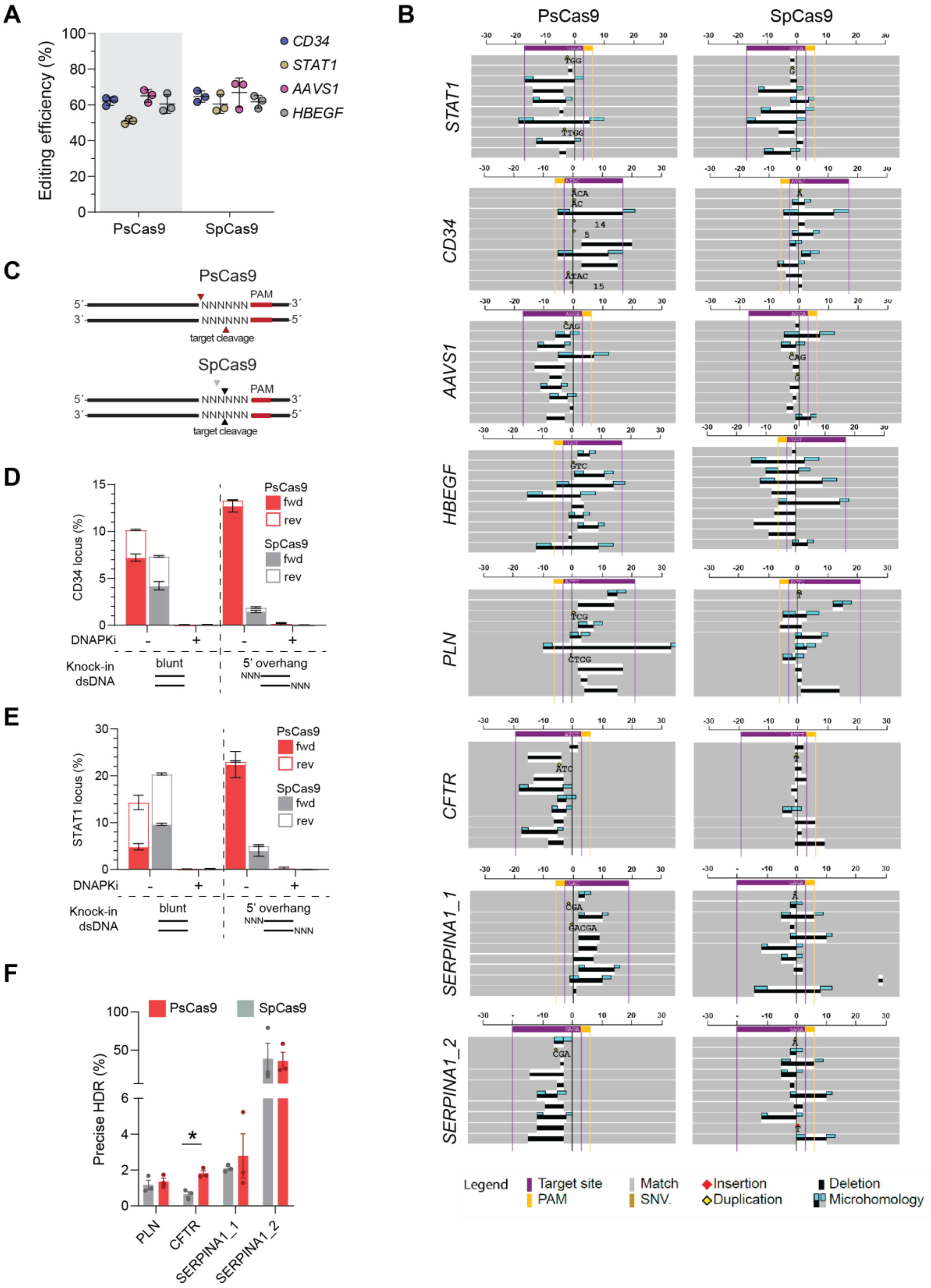

Supplementary Figure 5. Extended analysis of PsCas9 edits in the human genome.

a. Editing efficiency of the human genome by PsCas9 and SpCas9 on depicted loci upon plasmid transfection of HEK293T cells. The editing efficiency was assessed by CRISPResso2 analysis of NGS data and shown as the percentage modified reads in each sample. Data are presented as mean ± SD, n=3.

b. Visualization of the top ten most frequent mutations of PsCas9 and SpCas9 using the same sgRNA at selected target sites (*STAT1*, *CD34*, *AAVS1*, *EMX1a*, *HBEGF*, *PLN*, *CFTR* and *SERPINA1* with two different sgRNAs) analyzed by RIMA[55].

c. Schematic depicting the genomic DNA cutting pattern induced by PsCas9 (red) or SpCas9 (black/grey).

d-e. Genome insertion efficiency (knock-in, %) at *CD34* (d) or *STAT1* (e) by PsCas9 (red) and SpCas9 (grey) in HEK293T cells. Double-stranded DNA substrates used for the insertions are depicted below. Experiments were performed with or without DNA-PK inhibitor (DNA-PKi, M9831). The knock-in efficiency (reverse (empty) or forward (filled) direction) was calculated using NGS and shown as percentage of knock-in from total modified reads in each sample. Data are presented as mean ± SD, n=3.

f. Precise HDR-mediated genome insertion efficiency (%) at *PLN*, *CFTR*, *SERPINA1* (two independent sgRNAs) by PsCas9 (red) and SpCas9 (grey) in HEK293T cells. The precise HDR efficiency was calculated using NGS and shown as percentage of knock-in from total reads in each sample. A two-sided Student's t-test was performed to evaluate statistical significance (*P-value = 00.5317). Data are presented as mean ± SD, n=3.

Bestas et al. Supplementary Figure 6

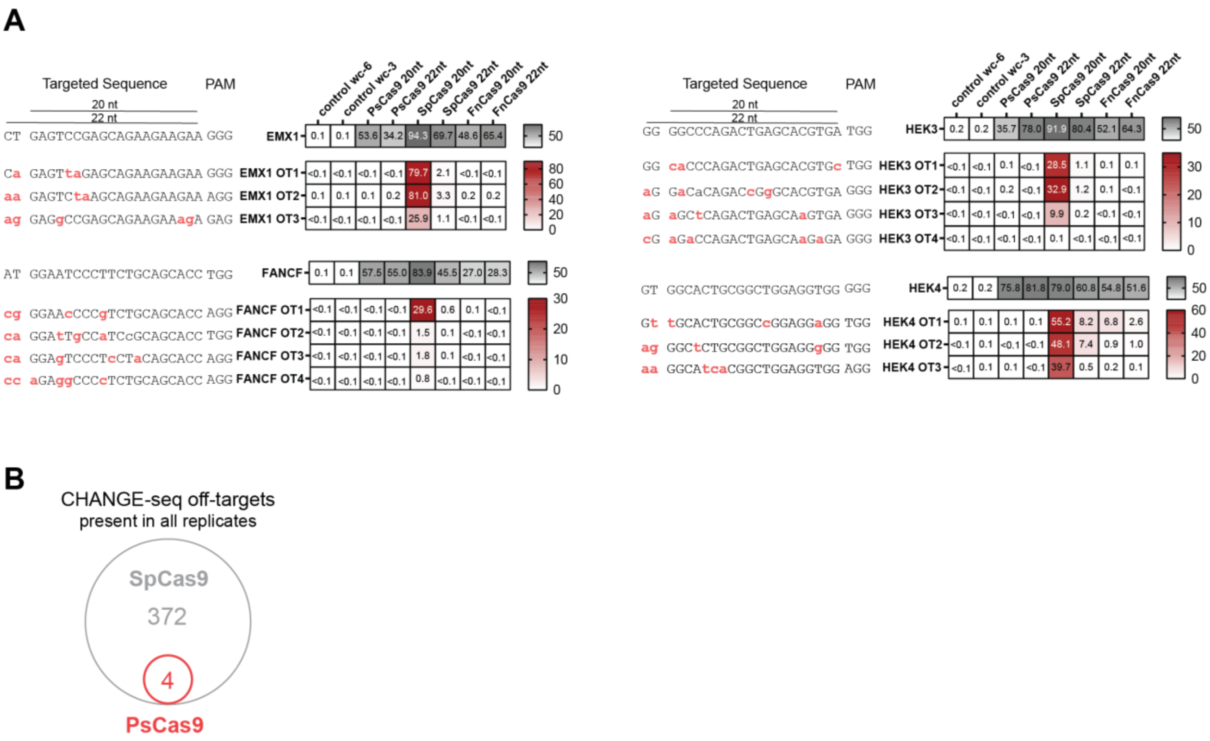

Supplementary Figure 6. Extended analysis of PsCas9 edits in the human genome.

a. Heatmap showing *EMX1*, *FANCF*, *HEK3* and *HEK4* editing and off-target editing by PsCas9, SpCas9 and FnCas9 with 20 nt and 22 nt spacer (right). The DNA on-target site and three to four off-target sites are shown on the left with mismatches highlighted in red. Control samples were untransfected and the editing efficiency was determined using NGS. Sequencing data was analyzed by CRISPResso2 and presented as percentages of modified reads in each sample. Data are presented as mean ± SD, n=3.

b. Chart showing the HEK4 CHANGE-seq off-target hits overlap between SpCas9 (20-nt spacer) and PsCas9 (22-nt spacer). Only hits that are present in all three replicates were considered (Supplementary Data Table 7).

Bestas et al. Supplementary Figure 7

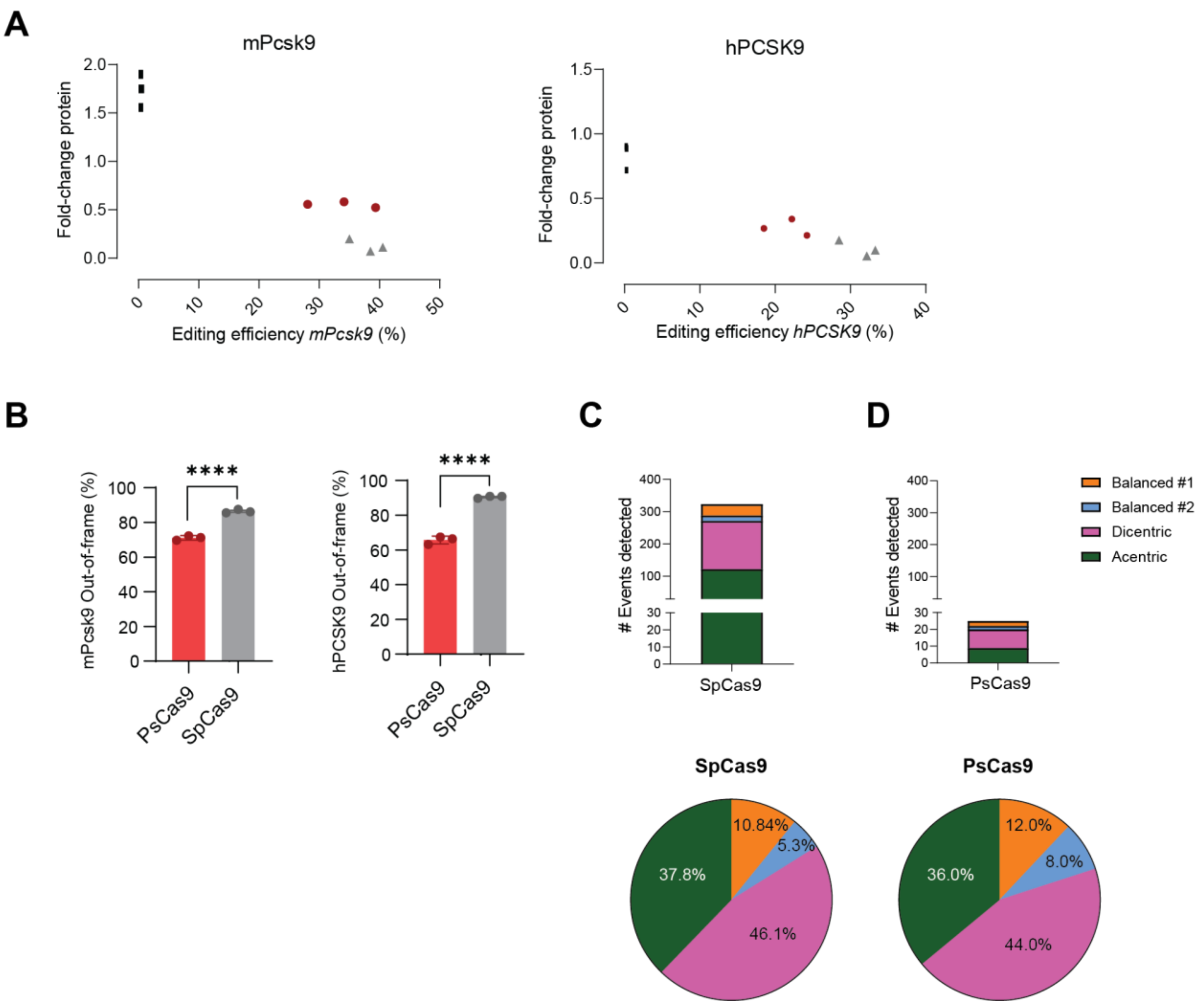

Supplementary Figure 7. Extended *in vivo* data.

a. Correlation of editing efficiency and reduction of plasma protein levels *in vivo*. Editing efficiencies at the mouse *Pcsk9* (left) and human *PCSK9* (right) loci were determined by targeted deep amplicon sequencing. Control samples are presented in black, PsCas9 in red and SpCas9 in grey. Changes in plasma protein levels were calculated from ELISA measurements of plasma samples taken prior to virus injection (baseline) and during termination. Note that control virus-treated animals showed an increase in mouse Pcsk9 plasma levels over the course of the study (represented as negative values of reduction).

b. Editing outcome at the on-target sites *in vivo*. The percentage of out-of-frame mutations induced at the mouse (left panel) and human *PCSK9* sites (right panel) as calculated from amplicon-sequencing results is shown. Graphs show

individual values for each mouse (n=3 independent animals), as well as mean ± SEM. Unpaired, two-tailed t-test was performed to evaluate statistical significance (****P-value ≤ 0.0001).

c-d. Bar plots (above) and pie charts (below) presenting color-coded types of translocation events detected in Duplex-seq data in the animals treated with SpCas9 (c) and PsCas9 (d).
