# Supplementary material for: A Type II-B Cas9 nuclease with minimized off-targets and reduced chromosomal translocations in vivo
Source: Nat Commun. 2023 Sep 6;14:5474. doi: 10.1038/s41467-023-41240-7 (PMC10482872; doi:10.1038/s41467-023-41240-7)
Supplement: Supplementary file 2 — Description of Additional Supplementary Files [file 41467_2023_41240_MOESM2_ESM.pdf]

Title: Supplementary Data 1.
Description: Output of the PILER-CR analysis of CRISPR repeats.

Title: Supplementary Data 2.
Description: RNA Sequences and DNA oligos used in the study.

Title: Supplementary Data 3.
Description: Protein sequences and DNA constructs used in the study.

Title: Supplementary Data 4.
Description: CRISPResso 2 parameters.

Title: Supplementary Data 5.
Description: Detailed description of Duplex-Seq.

Title: Supplementary Data 6.
Description: Enrichment probes and VariantCalling analysis regions.

Title: Supplementary Data 7.
Description: CHANGE-seq data.
