# Supplementary material for: A Type II-B Cas9 nuclease with minimized off-targets and reduced chromosomal translocations in vivo
Source: Nat Commun. 2023 Sep 6;14:5474. doi: 10.1038/s41467-023-41240-7 (PMC10482872; doi:10.1038/s41467-023-41240-7)
Supplement: Supplementary file 3 — Reporting Summary [file 41467_2023_41240_MOESM3_ESM.pdf]

nature portfolio

Corresponding author(s): NCOMMS-22-40165B

Last updated by author(s): 2023/08/13

Reporting Summary

Nature Portfolio wishes to improve the reproducibility of the work that we publish. This form provides structure for consistency and transparency in reporting. For further information on Nature Portfolio policies, see our Editorial Policies and the Editorial Policy Checklist.

Statistics

For all statistical analyses, confirm that the following items are present in the figure legend, table legend, main text, or Methods section.

n/a	Confirmed	
[ ]	[x]	The exact sample size (*n*) for each experimental group/condition, given as a discrete number and unit of measurement
[x]	[ ]	A statement on whether measurements were taken from distinct samples or whether the same sample was measured repeatedly
[ ]	[x]	The statistical test(s) used AND whether they are one- or two-sided<br>*Only common tests should be described solely by name; describe more complex techniques in the Methods section.*
[x]	[ ]	A description of all covariates tested
[ ]	[x]	A description of any assumptions or corrections, such as tests of normality and adjustment for multiple comparisons
[ ]	[x]	A full description of the statistical parameters including central tendency (e.g. means) or other basic estimates (e.g. regression coefficient) AND variation (e.g. standard deviation) or associated estimates of uncertainty (e.g. confidence intervals)
[ ]	[x]	For null hypothesis testing, the test statistic (e.g. *F*, *t*, *r*) with confidence intervals, effect sizes, degrees of freedom and *P* value noted<br>*Give P values as exact values whenever suitable.*
[x]	[ ]	For Bayesian analysis, information on the choice of priors and Markov chain Monte Carlo settings
[x]	[ ]	For hierarchical and complex designs, identification of the appropriate level for tests and full reporting of outcomes
[x]	[ ]	Estimates of effect sizes (e.g. Cohen's *d*, Pearson's *r*), indicating how they were calculated

Our web collection on statistics for biologists contains articles on many of the points above.

Software and code

Policy information about availability of computer code

Data collection	Data collection for Cas9 II-B identification from human gut microbiome:<br>A search was performed using hmmscan (version 3.1b2) from the HMMER package using the profile TIGR03031 from the TIGRFAM collection of Hidden Markov Models as the query. The TIGR03031 hmm model, designated as IPR013492 in the Interpro database, includes proteins of CRISPR system subtype II-B RNA-guided endonucleases. The set of proteins searched was from the catalogue of microbial genes in the integrated genome catalogue (IGC) from the human gut microbiome. The contigs from the metagenomic assembly were obtained for the hits and CRISPRs were identified with pilercr43. To determine the taxonomic origin of MH0245 Cas9, BLAST searches were performed.<br><br>sgRNA design:<br>We used AstraZeneca proprietary software as an in silico tool, which was developed based on Wellcome Trust Sanger Institute`s codebase (WGE: http://www.sanger.ac.uk/htgt/wge/).<br><br>Western Blot:<br>Odyssey Imager Li-COR Biosciences GmbH<br><br>ELISA:<br>PHERAstar FSX - Microplate Reader
Data analysis	Statistical analysis:<br>Graphpad Prism 6 or 9 (GraphPad Software, Inc)

March 2021

NGS analysis:
Amplicon-Sequenci
De-multiplexing - Bcl2Fastq software version 2.20.0.422 (Illumina)
Analysis: CRISPResso version 2.1.1 (Clement et al. 2019, Nat. Biotech. https://www.nature.com/articles/s41587-019-0032-3), RIMA (Taheri-Ghahfarokhi et al., 2018, NAR, https://academic.oup.com/nar/article/46/16/8417/5055824?login=true), Python3, R version 3.4.2,
for PAM analysis/ dsDNA integration FASTQ files were analyzed using a Perl (version 5.26.1) implementation of the Matlab script described previously (see e.g. Li et al., 2021 Nat. Comm. https://www.nature.com/articles/s41467-020-20810-z)
Duplex-Sequencing:
bcbio611 nextgen variant calling pipeline v1.2.7; BWA-MEM (reference alignment); fgbio (Duplex UMI processing, read grouping, collapsing); CRISPResso version 2.1.1

ddPCR:
QuantaSoft (Bio-Rad)

In vitro kinetics:
R 3.6.3 with RStudio + tidyverse, fragman packages

PAM identification:
Position frequency matrix, R 3.6.3 + ggseqlogo

CHANGE-seq:
The sequenced data was analysed using the previously published CHANGE-seq analysis pipeline (https://github.com/tsailabSJ/changeseq) with minor modifications. The pipeline was run with the following parameters: read_threshold: 4, window_size: 3, mapq_threshold: 50, start_threshold: 1, gap_threshold: 3, mismatch_threshold: 6, search_radius: 30, merged_analysis: False, target sequence: GGCACTGCGGCTGGAGGTGGNGG. Reads with MAPQ = 0 were included in the analysis alongside those passing the MAPQ threshold defined in the parameters, in order to nominate putative off-targets located in non-uniquely mappable regions.

For manuscripts utilizing custom algorithms or software that are central to the research but not yet described in published literature, software must be made available to editors and reviewers. We strongly encourage code deposition in a community repository (e.g. GitHub). See the Nature Portfolio guidelines for submitting code & software for further information.

Data

Policy information about availability of data

All manuscripts must include a data availability statement. This statement should provide the following information, where applicable:
- Accession codes, unique identifiers, or web links for publicly available datasets
- A description of any restrictions on data availability
- For clinical datasets or third party data, please ensure that the statement adheres to our policy

All raw and/or analyzed data are available from the corresponding author upon request.

Human research participants

Policy information about studies involving human research participants and Sex and Gender in Research.

Reporting on sex and gender	n/a
Population characteristics	n/a
Recruitment	n/a
Ethics oversight	n/a

Note that full information on the approval of the study protocol must also be provided in the manuscript.

Field-specific reporting

Please select the one below that is the best fit for your research. If you are not sure, read the appropriate sections before making your selection.

- [x] Life sciences
- [ ] Behavioural & social sciences
- [ ] Ecological, evolutionary & environmental sciences

For a reference copy of the document with all sections, see nature.com/documents/nr-reporting-summary-flat.pdf

Life sciences study design

All studies must disclose on these points even when the disclosure is negative.

Sample size	Sample sizes were determined based on literature precedence for genome editing experiments (e.g. Peterka et al., 2022 Nat. Comm.<br>Animal experiments: no sample size calculation was performed. Sample size was determined as generation of triple independent samples for comparisons between groups that is sufficient to perform statistical tests.
Data exclusions	No data was excluded in cell and in vitro experiments.<br>Each animal treatment group contained 4 animals. Only three animals of each group were subjected for Duplex-Seq analysis. Animals with highest editing efficiency were selected.
Replication	All cell data and in vitro experiment were independently repeated at least once as specified in figure legends.<br>Animal experiments: Three animals were included per group.
Randomization	Mammalian cells were cultured under identical conditions, no randomization was used.<br>Animals were randomized based on their weights measured prior to the experiments.
Blinding	Mammalian cells used in this study were grown under identical conditions, no blinding was used.<br>No blinding was used for animal studies.

Reporting for specific materials, systems and methods

We require information from authors about some types of materials, experimental systems and methods used in many studies. Here, indicate whether each material, system or method listed is relevant to your study. If you are not sure if a list item applies to your research, read the appropriate section before selecting a response.

Materials & experimental systems

n/a	Involved in the study
[ ]	[x] Antibodies
[ ]	[x] Eukaryotic cell lines
[x]	[ ] Palaeontology and archaeology
[ ]	[x] Animals and other organisms
[x]	[ ] Clinical data
[x]	[ ] Dual use research of concern

Methods

n/a	Involved in the study
[x]	[ ] ChIP-seq
[x]	[ ] Flow cytometry
[x]	[ ] MRI-based neuroimaging

Antibodies

Antibodies used	primary antibodies: Monoclonal ANTI-FLAG® M2, Clone M2 (F1804, Sigma-Aldrich; dilution 1:2000), GAPDH (D16H11) XP® Rabbit mAb #5174 as an internal control (D16H11, Cell Signaling Technology; dilution 1:10000).<br>secondary antibodies (all 1:10000 dilution: goat anti-mouse 800CW, and goat anti-rabbit 680 (all from LI-COR Biosciences GmbH)
Validation	All antibodies listed above were purchased commercially and validated by suppliers for their performance.

Eukaryotic cell lines

Policy information about cell lines and Sex and Gender in Research

Cell line source(s)	HEK293T (GenHunter Corporation, Q401)
Authentication	HEK293T were authenticated using STR profiling and were >80% identity matched.
Mycoplasma contamination	HEK293T were negatively tested for Mycoplasma contamination.

Commonly misidentified lines (See ICLAC register)	No commonly misidentified lines were used.

Animals and other research organisms

Policy information about studies involving animals; ARRIVE guidelines recommended for reporting animal research, and Sex and Gender in Research

Laboratory animals	Humanized PCSK9 knock-in mouse model is derived from C57BL/6N mice, male, 5 month old.<br>C57BL/6N mice (Charles River, Sulzfeld, Germany)<br>Generation of humanized PCSK9 knock-in mouse model is described in the publication of Carreras et al. , 2019, BMC Biol. (https://www.ncbi.nlm.nih.gov/pmc/articles/PMC6334452/).
Wild animals	No wild animals were used in this study.
Reporting on sex	No sex based analysis has been performed. We only used males to keep results consistent between different analysis.
Field-collected samples	No field-collected samples were generated or used in this study.
Ethics oversight	All mouse experiments were approved by the AstraZeneca internal committee for animal studies and the Gothenburg Ethics Committee for Experimental Animals (license numbers: 162-2015+ and 2194-2019) compliant with EU directives on the protection of animals used for scientific purpose.

Note that full information on the approval of the study protocol must also be provided in the manuscript.
