# Supplementary material for: A Type II-B Cas9 nuclease with minimized off-targets and reduced chromosomal translocations in vivo
Source: Nat Commun. 2023 Sep 6;14:5474. doi: 10.1038/s41467-023-41240-7 (PMC10482872; doi:10.1038/s41467-023-41240-7)
Supplement: Supplementary file 9 — Supplementary Data 5 [file 41467_2023_41240_MOESM9_ESM.docx]

**Duplex sequencing (detailed description)**

**Capture Panel Design:** Potential off-target regions (n=79) had been previously determined by CIRCLE-seq (PMID: 30209390). For the two on-target regions and all potential off-target regions, four capture probes each were designed (40 bases upstream, 20 bases upstream, 20 bases downstream, and 40 bases downstream of the cut site). Additionally, probes were designed for 40 additional sites which served as control regions. During probe design and QC, a subset of proposed baits were determined to have high potential for off target capture effects and were therefore excluded from the final capture panel. For potential off-target regions, four regions were completely excluded and ten regions had less than four capture probes designed. For the control regions, three regions were completely excluded. Final panel consisted of 327 probes after removal of the poor-performing probes.

**Genomic Libraries:** DNA from each mouse was QC’ed by TapeStation gDNA assay before shearing on a Covaris M220 acoustic shearing instrument with settings targeting 300 base pair fragments. Post shearing, each of the sheared DNA samples were split into three replicates. All samples were then proceeded through the xGen Prism Library Preparation kit from IDT following the standard protocol with 12 cycles of amplification. Genomic libraries were quantified by the TapeStation D1000 assay.

**Target Capture:** Replicate libraries were pooled together pre-capture and proceeded through the xGen Hybridization and Wash kit from IDT following the standard protocol with 15 cycles of amplification post-capture one. After capture one was complete, the samples were processed through hybridization capture a second time to ensure high on target coverage. All pooled libraries proceeded through the xGen Hybridization and Wash kit from IDT following the standard protocol with 7 cycles of amplification post-capture two. Both captures utilized the previously described capture panel, TS Mix xGen Universal Blockers and Mouse Cot-1 DNA. Capture libraries were quantified by the TapeStation D1000 assay.

**Sequencing and Informatics:** All capture libraries were pooled equimolarly and sequenced on a NovaSeq 6000 instrument, using an S2 flowcell. Sequencing data was processed with bcbio-nextgen variant calling pipeline v1.2.7 utilizing a custom genome reference based on mm10 genome with the addition of the PCSK9 transgene content. Reads were aligned to the reference with bwa-mem, duplex UMI processing, read grouping and collapsing were performed with fgbio, variants were called with vardict and annotated with snpEff.

**Assay Efficiency and Depth Achieved**

| Sample ID | Treatment | input (ng) | Input (GE) | Average Depth (X) | Assay Efficiency |
| --- | --- | --- | --- | --- | --- |
| 2106 | SpOT-ON+gMH | 346 | 128020 | 12310 | 9.6% |
| 2112 | SpOT-ON+gMH | 350 | 129500 | 14309 | 11.0% |
| 2126 | SpOT-ON+gMH | 392 | 145040 | 14708 | 10.1% |
| 1487 | SpCas9+GFP | 336 | 124320 | 12458 | 10.0% |
| 1512 | SpCas9+GFP | 283 | 104710 | 11561 | 11.0% |
| 1524 | SpCas9+GFP | 355 | 131350 | 12750 | 9.7% |
| 1484 | SpCas9+gMH | 336 | 124320 | 11981 | 9.6% |
| 1493 | SpCas9+gMH | 283 | 104710 | 11682 | 11.2% |
| 1518 | SpCas9+gMH | 288 | 106560 | 11155 | 10.5% |

**Filtering strategy**

1. Bin all VarDict calls from each experiment arm separately (Control SpCas9+GFP, MHCas9, SpCas9).
2. Within each bin, count occurrence of unique mutations as well as variant depth (info.VD).
3. Assign ‘VD<3’ flag to samples that have variant depth less than 3 AND with a single mutation count. Mutations flagged as ‘VD<3’ are single occurrence variants and cannot be considered to be high fidelity.
4. All other variants assigned the ‘VD>=3’ flag, denoting that the variant has at least 3 reads in one sample or was identified in more than one sample in the bin.

Translocation Analysis

All discordant inter-chromosomal reads were parsed out of bam files. A series of filters were applied:

- Remove unmapped reads (FLAG = 77 or 141)
- Remove reads with poor mapping quality (MAPQ<60)
- Remove reads with unknown placement (RNAME/RNEXT includes chrUn)
- Remove reads with low number of supporting pre-consensus reads (cD<20)
- Remove reads with large edit distance to reference must be (NM>1)
- Remove reads with low alignment score (AS<35)
